# Long-term efficacy and safety of anticoagulant for cavernous transformation of the portal vein cirrhotic patient with extrahepatic portal vein obstruction

**DOI:** 10.1186/s12959-023-00449-8

**Published:** 2023-01-11

**Authors:** Yongjie Zhou, Zhiquan Zhuang, Tianzhu Yu, Wen Zhang, Jingqin Ma, Jiaze Yu, Zhiping Yan, Jianjun Luo

**Affiliations:** 1 Department of Interventional Radiology, Zhongshan Hospital, Fudan University, Shanghai, China; 2 Shanghai Institution of Medical Imaging, Shanghai, China; 3 National Clinical Research Center for Interventional Medicine, Zhongshan Hospital, Fudan University, Shanghai, China; 4 Department of Interventional Radiology, Xiamen Branch, Zhongshan Hospital, Fudan University, Shanghai, China; 5 Center for Tumor Diagnosis and Therapy, Jinshan Hospital, Fudan University, Shanghai, China

**Keywords:** Portal vein thrombosis, Cavernous transformation of the portal vein, Extrahepatic portal vein obstruction, Cirrhosis, Warfarin, Hepatic decompensation

## Abstract

**Background/aims:**

Cavernous transformation of the portal vein (CTPV) in cirrhotic patients with extrahepatic portal vein obstruction (EHPVO) was a relatively rare disease and had no consensus on the treatment. Our study aimed to explore the value of anticoagulation with warfarin treatment for CTPV cirrhotic patients with EHPVO.

Methods: From January 2015 to December 2019, the clinical characteristics of cirrhotic patients who were diagnosed as CTPV with EHPVO were retrospectively analyzed. Eligible patients were distributed into the anticoagulation group (*n* = 46) and control group (*n* = 38). The change of portal vein thrombosis, hepatic decompensation, survival and adverse events were evaluated between the two groups.

**Results:**

The median follow-up of our patients was 51 months in the anticoagulation group and 44 months in the control group. The progress rate of the portal vein was higher in patients from the control groups (*n* = 12) than in patients from the anticoagulation group (*n* = 4, *p* = 0.008). There was no significant difference between the partial recanalization rate and stable rate between the two groups. Patients in anticoagulation group developed less hepatic decompensation than those in control group (13.0% vs 34.2%, *p* = 0.021). The Kaplan-Meier curve showed that patients in the anticoagulation group had a better prognosis than patients in the control group (*P* < 0.022). There were no serious complications due to warfarin treatment.

**Conclusion:**

For CTPV cirrhotic patients with EHPVO, anticoagulation with warfarin treatment was effective and safe. Anticoagulants could prevent portal vein thrombosis progression, hepatic decompensation and death. In addition, our results showed little benefit of anticoagulants on thrombosis recanalization.

**Supplementary Information:**

The online version contains supplementary material available at 10.1186/s12959-023-00449-8.

## Introduction

Portal vein thrombosis (PVT) was a frequent event in cirrhotic patients, especially at decompensated stage [1]. The prevalence of PVT ranges from 7.4 to 16.4% in liver cirrhosis [2–4]. Cavernous transformation of the portal vein (CTPV) was dilated and tortuous collateral plexus in the hepatic hilar region and was primarily secondary to PVT. In general, portal hypertension due to extrahepatic portal vein obstruction (EHPVO) could result in the occurrence of CTPV. In 1869, Balfour and Stewart first reported the clinical characteristics and pathology of CTPV [5]. As a relatively rare disease, CTPV could exacerbate portal hypertension, leading to esophagogastric variceal bleeding, refractory ascites，infectious peritonitis and liver failure, especially combined EHPVO.

There was only consensus on chronic non-cirrhotic extrahepatic portal vein obstruction with EHPVO [6]. Regretfully, no guideline or consensus on CTPV cirrhotic patients EHPVO was established due to low incidence and little research. The current therapy was mainly based on the clinician’s experience. CTPV with EHPVO was a relative contraindication of liver transplant, because disorganized veins can increase the risk of surgery. Anticoagulation, transjugular intrahepatic portosystemic shunt (TIPS), endoscopy treatment and portal vein stenting were reported in the treatment of CTPV with cirrhosis, with unsatisfactory efficacy [7–10]. Theoretically, TIPS and portal vein stenting could recanalize the portal vein which results in decreased portal pressure and better liver function. However, the presence of EHPVO increased the operational difficulties of this treatment. Endoscopy treatment, including endoscopic variceal ligation (EBL) and cyanoacrylate injection, was recommended for the management of esophagogastric varices. The persistence of portal hypertension could lead to decompensated cirrhosis and cause new esophagogastric varices. Anticoagulation is the cornerstone of the treatment for PVT [1, 9]. Several studies [10–13] showed anticoagulation treatment could increase the recanalization rate of the portal vein in cirrhosis patients and decrease the risk of esophagogastric variceal bleeding. However, CTPV patients with EHPVO were excluded from theses research. The efficacy of anticoagulation treatment for CTPV patients with EHPVO was urgent to be investigated.

The purpose of our study was to explore the value of anticoagulation treatment for CTPV cirrhotic patients with EHPVO.

## Materials and methods

### Patients selection

From January 2015 to December 2019, the clinical characteristics of cirrhotic patients diagnosed as CTPV cirrhotic patients with EHPVO in Zhongshan hospital were retrospectively analyzed. Our study protocol was approved by the Ethics Committee and Institutional Review Board of Zhongshan Hospital of Fudan University. Written informed consent was obtained from eligible patients.

Patients who met with following criteria were considered in our study: (1) cirrhosis was diagnosed by clinical manifestation, laboratory test, imaging study and liver biopsies. (2) CTPV patients with EHPVO were diagnosed by contrast-enhanced Computed Tomography (CT) and contrast-enhanced Magnetic Resonance Imaging (MRI); (3). age between 18 and 75 years. (4) adequate liver and renal function: Child-Pugh score ≤ 9, aspartate aminotransferase (ALT) and alanine aminotransferase (AST) < 5 × upper limits of normal, alkaline phosphatase < 4 × upper limits of normal, total bilirubin <51umol/L, serum creatinine ≤115 umol/L; The exclusion conditions were as following: (1) hepatocellular carcinoma or other extrahepatic tumors; (2) a history of TIPS or portal vein stent implantation treatment. (3). pregnancy and breastfeeding; (4) uncontrolled systemic infection or sepsis.

**Fig. 1:**
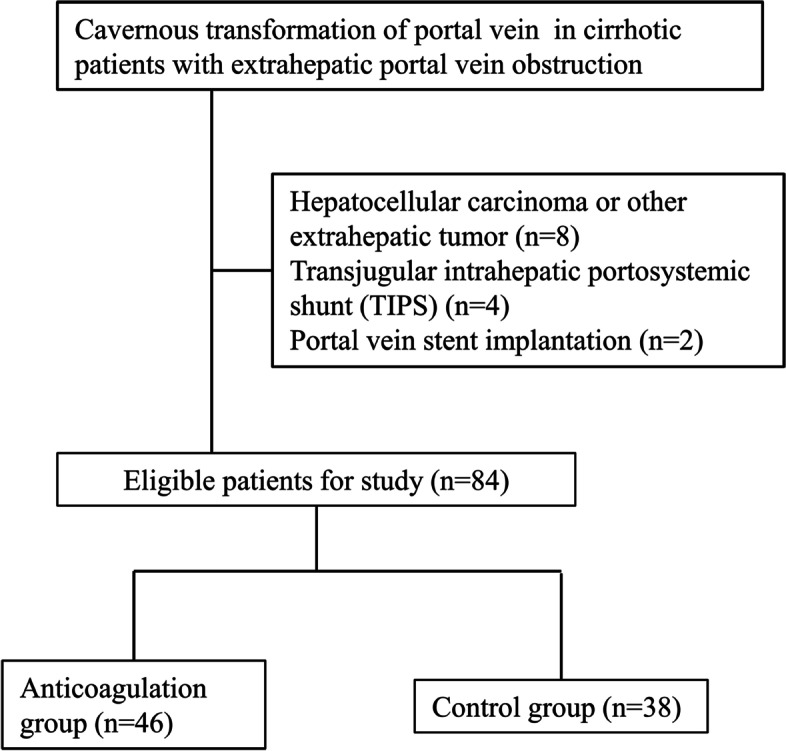
The flow chart of the enrollment of eligible cavernous transformation of portal vein cirrhotic patients with extrahepatic portal vein obstruction

Based on the inclusion and exclusion criteria, 46 patients who received warfarin treatment were selected for the anticoagulation group and 38 patients who were not anticoagulated were allowed into the control group. The flow chart of enrollment was shown in Fig. 1. Patients were followed until death, or turning other treatments (TIPS, thrombolysis, endoscopy therapy), or the deadline of our study (June, 2022).

**Table 1:** The clinical characteristics of eligible patients

Variables		Anticoagulation group (*n* = 46)	Control group (*n* = 38)	*P* value
Age (years)		47.19 ± 15.79	49.61 ± 11.11	0.431
Gender	Male	32	26	0.91
	Female	14	12	
Etiology	HBV	34	28	0.995
	HCV	4	3	
	Alcohol	3	3	
	Other	5	4	
Platelets (×109/l)		89.89 ± 58.09	88.34 ± 54.17	0.901
Serum total bilirubin (μmol/l)		20.12 ± 11.51	18.34 ± 8.42	0.43
Serum albumin (g/l)		35.46 ± 5.49	36.73 ± 5.12	0.276
Serum creatinine (μmol/l)		70.57 ± 13.73	68.58 ± 16.12	0.55
PT(s)		14.68 ± 2.27	14.07 ± 1.83	0.192
INR		1.33 ± 0.21	1.31 ± 0.16	0.65
CHILD score		5.85 ± 0.92	5.71 ± 0.89	0.493
CHILD grade	A	36	31	0.706
	B	10	7	
MELD score		7.76 ± 3.28	7.28 ± 3.97	0.541
Esophagogastric varices	Present	26	21	0.908
	Absent	20	17	
Previous variceal bleeding	Present	18	16	0.782
	Absent	28	22	
Ascite	Present	10	7	0.706
	Absent	36	31	
Splenectomy	Present	16	13	0.956
	Absent	30	25	
Vessel involvement	MPV only	22	20	0.839
	MPV+ SV only	14	12	
	MPV+ SMV only	8	4	
	MPV+ SV + SMV	2	2	
Follow up time		51	44	0.099

*HBV *Hepatitis B virus; HCV:hepatitis C virus; PT: prothrombin time;INR, international normalized ratio; MELD, model for end-stage liver; MPV: main portal vein; SV: spleen vein; SMV: superior mesenteric vein

### Anticoagulation protocol

Patients the in the anticoagulation group received warfarin treatment at an initial dose of 2.5 mg daily. The international normalized ratio (INR) was used to evaluate the effect of warfarin, with a target of 1.8–2.5. The dosage of warfarin was carefully adjusted through the increase or decrease of 0.625 mg (1/4 piece) to achieve the target of INR. The INR was initially monitored every week, and the follow time of INR could prolong until 1 month if the INR was stable in 1.8–2.5. Patients in the control group were not administered any other anticoagulation treatment.

#### The efficacy of anticoagulation treatment

The portal vein thrombosis recanalization included complete and partial resolution. The complete resolution was defined as the absolute disappearance of main portal vein thrombosis, and the partial resolution was defined as at least a 50% reduction of the thrombosis on the cross-section, without the appearance of new thrombosis. If the thrombosis increased more than 30% on the cross-section or extended into a new segment of the spleen vein and superior mesenteric vein, thrombosis met the progression criteria. Stable thrombosis was stated if thrombosis was not conforming to complete resolution, partial resolution or progression.

Hepatic decompensation was defined as a composite incidence that compromised one or several of the following event: variceal bleeding, development of ascites, hepatic encephalopathy, and infectious peritonitis. The survival time was calculated from diagnosis at admission to death.

### Statistical analysis

Continuous and categorical variables were expressed as mean ± SD and percentage, respectively. Independent t-test or Mann-Whitney U test were performed to compare continuous variables, while the Chi-square test or Fisher’s exact test were used for categorical variables. The Kaplan–Meier curves and log-rank test were performed to compare the hepatic decompensation and overall survival between two groups. The univariate and multivariate cox regression method was conducted to identify the predictor for hepatic decompensation and overall survival. *P* value < 0.05 signified a significant difference. Visualization and analysis were performed with GraphPad Prism version 6.0 (GraphPad Software, Inc., La Jolla, CA) and R software (4.0.4).

## Results

### The clinical characteristics of eligible patients

From January 2015 to December 2019, the CTPV cirrhotic patients with EHPVO who were diagnosed in Zhongshan hospital were preliminarily screened in our study. Finally, based on the inclusion and exclusion criteria, 46 eligible patients were included in the anticoagulation group, and 38 patients were selected into the control groups. The clinical characteristics of these patients at the time of admission were presented in Table 1. there were no significant differences in clinical characteristics between the two groups. The mean age of patients was 47.19 ± 15.79 and 49.61 ± 11.11, respectively, and the primary etiology was HBV, followed by HCV and alcohol. The median follow-up of our patients was 51 months in the anticoagulation group and 44 months in the control group.

### Efficacy anticoagulation treatment

The mean dose of warfarin in the anticoagulation group was 1.35 ± 2.12, and the mean value of the international normalized ratio (INR) was 2.12 ± 3.21 (rang 1.53 to 2.32). Overall, 44 patients (95.7%) fulfilled the INR criteria of 1.8–2.5, except 2 patients because of gingival bleeding and diarrhea. These symptoms disappeared in patients after the reduction of warfarin. 46 patients received warfarin treatment until death and the deadline of follow-up time. The rest of the 8 patients stopped warfarin treatment due to the following reason: hematemesis or melena (*n* = 1), transition to rivaroxaban treatment (*n* = 3), and no special reason (n = 1).

After the evaluation of portal vein thrombosis through a contrast-enhanced CT scan during follow-up. In the anticoagulation group, 8 patients (17.4%) achieved partial recanalization, whereas only 2 patients (5.3%) showed partial recanalization in the control group (*P* = 0.088, Table 2). The progression rate of portal vein was higher in patients from the control group (*n* = 12) than in patients from the anticoagulation group (*n* = 4, *p* = 0.008). Thirty-six patients (75%) in the anticoagulation group and 24 patients (63.2%) in the control group showed stable, with no statistical difference (*p* = 0.235).

**Table 2:** The changes of thrombosis in two groups

Variables	Anticoagulation group (*n* = 46)	Control group (*n* = 38)	*P* value
Complete recanalization	0	0	–
Partital recanalization	8 (17.4%)	2 (5.3%)	0.088
Stable	36 (75%)	24 (63.2%)	0.235
Progress	4 (8.7%)	12 (31.6%)	0.008*

^✱^ indicates significance of P<0.05

#### The outcome of hepatic decompensation and survival

During the follow-up time, hepatic decompensation occurred in 26 patients, including 6 patients (13.0%) in the anticoagulation group and 13 patients (34.2%) in the control group (*p* = 0.021, Table 3). The summary of hepatic decompensation in two groups was shown in Table 3. The frequent event of hepatic decompensation was variceal rebleeding, the incidence rate of which was lower in the anticoagulation group (*n* = 3) than in the control group (*n* = 8). Endoscopic band ligation (EBL) and cyanoacrylate injection treatment was performed on 6 patients with variceal bleeding, including 2 patients in the anticoagulation group and 6 patients in the control group. TIPS treatment was successfully achieved in 3 patients, involving 1 patient in the anticoagulation group and 2 patients in the control group. After the operation, the patient in the anticoagulation group continued on warfarin therapy. Our cox regression model showed that *anticoagulation* was the only predictor for hepatic decompensation (Table 4).

**Table 3:** The clinical outcomes between two groups

Variables	Anticoagulation group (n = 46)	Control group (*n* = 38)	*P* value
Hepatic decompensation	6 (13.0%)	13 (34.2%)	0.021*
Variceal bleeding	3 (6.5%)	8 (21.1%)	0.046*
Ascite	2 (5.5%)	2 (4.3%)	0.845
Hepatic encephalopathy	1 (2.1%)	1 (2.6%)	0.891
Infectious peritonitis	0	2 (5.3%)	–
Death	3 (6.5%)	9 (23.7%)	0.025*
Liver failure	1 (2.1%)	4 (10.5%)	0.097
Hemorrhoea	0	3 (7.9%)	–
Infectious peritonitis	1 (2.1%)	2 (5.3%)	0.425
Renal failure	1 (2.1%)	0	–

^✱^ indicates significance of *P*<0.05

**Table 4:** Univariate and multivariate analysis for hepatic decompensation and survival

Variables	Univariate analysis	*P* value	Multivariate analysis	*P* value
	HR(95%CI)		HR(95%CI)	
Hepatic decompensation				
Age (years)	1.268 (1.124–1.524)	0.024		
Previous variceal bleeding	2.136 (1.574–2.942)	0.032		
Vessel involvement	1.846 (1.348–2.367)	0.042		
Anticoagulation treatment	0.845 (0.649–0.923)	0.002	0.765 (0.523–0.918)	0.028
Survival				
Previous variceal bleeding	1.587 (1.291–1.957)	0.026		
Anticoagulation treatment	0.726 (0.543–0.946)	0.012	0.821 (0.643–0.926)	0.018

**Fig. 2:**
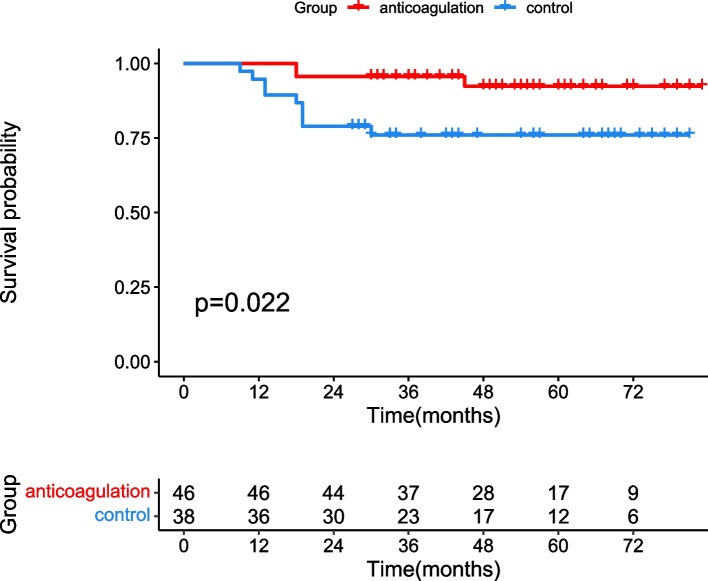
Kaplan-Meier curve evaluated the prognosis of patients between the anticoagulation group and the control group

Overall, 3 patients (6.5%) in the anticoagulation group and 9 patients (23.7%) in the control group died (*p* = 0.025). The Kaplan-Meier curve showed that patients in the anticoagulation group had a better prognosis than patients in the control group (*P* < 0.022, Fig. 2). The main cause of death was liver failure (1 patient in the anticoagulation group and 4 patients in the control group, p = 0.025). Death due to hemorrhage occurred in three patients from the control group. One patient (2.1%) in the anticoagulation group and two patients (5.3%) in the control group died because of infectious peritonitis. Renal failure contributed to death in 1 patient (2.1%) in the anticoagulation group. *Anticoagulation* was identified as the only factor for survival by using cox regression model (Table 4).

### Safety of anticoagulation treatment

During the follow-up time, there were no serious complications due to warfarin treatment. Two patients decreased the dosage of warfarin because of gingival hemorrhage and urticaria, respectively. Transit nausea occurred in 3 patients, and 2 patients developed transit diarrhea (Supplement Table S1).

## Discussion

CTPV with EHPVO in the cirrhotic patient was a relatively rare disease but could result in serious complications, such as esophagogastric variceal bleeding, recurrent ascites and hepatic failure. There was no consensus on the treatment of PVT with EHPVO. Anticoagulation treatment was recognized as the basic treatment for PVT in non-tumor patients [1, 9]. However, CTPV with EHPVO was always excluded from the studies which explored the effect of anticoagulation treatment for PVT. As far as we know, our study is firstly to explore the value of anticoagulation for CTPV cirrhotic patients with EHPVO. Our study showed that anticoagulation treatment could significantly prevent the progression of PVT and occurrence of hepatic decompensation, and *improve prognosis*, although had little effect on recanalization of main portal vein thrombosis.

CTPV was always secondary to long-term portal vein thrombosis and portal vein occlusion, and was considered to be an irreversible condition. One randomized controlled trial [13] showed that *nadroparin-warfarin* sequential anticoagulation could achieve a 62.5% recanalization rate (complete or partial) in cirrhotic patients with portal vein thrombosis. One systematic review and meta-analysis [12] performed by *Loffredo* demonstrated that patients treated with anticoagulation treatment had a higher recanalization rate compared with patients who did not accept anticoagulants (71% vs 42%, *P* < 0.0001). Regrettably, little recanalization rate was shown in CTPV cirrhotic patients with EHPVO who received anticoagulation therapy. Only several cases [7, 11] were reported, which showed that CTPV *was recanalized* after long-term anticoagulation treatment. Our results indicated that anticoagulants had little effect on portal thrombosis recanalization, but could prevent progression and maintain stability. We speculate that the following reasons could explain it. CTPV with EHPVO always had a long time course of the disease and was characterized by *fibrous thrombosis* and disruption of blood flow, which was difficult to be reversed by anticoagulant, especially with spleen and mesenteric venous thrombosis. Warfarin prevented thrombosis progression and *maintained* blood flow in the collateral circulation around the portal vein by adjusting the balance of hemostasis and anticoagulation.

Theoretically, recanalization of the portal vein achieved the restoration of blood flow to the liver and relieved portal hypertension, which avoided hepatic decompensation. A randomized controlled trial conducted by Zhou et [13] demonstrated that the Child-Pugh score and albumin level of cirrhotic patients with portal vein thrombosis were increased after six months of warfarin treatment. Our results indicated warfarin could prevent hepatic decompensation for CTPV cirrhotic patients with EHPVO, although had little effect on portal vein thrombosis recanalization. The unobstructed blood flow of cavernous transformation around the main portal vein was maintained by warfarin and prevented portal hypertension and ensured adequate hepatic blood perfusion, which decreased the incidence of hepatic decompensation. The Kaplan-Meier curve indicated patients in the anticoagulation group had a better prognosis. The primary cause of this death was correlated with hepatic decompensation, indicating a decrease in portal pressure and adequate blood flow to the liver could prevent hepatic decompensation and further increase the prognosis for CTPV cirrhotic patients with EHPVO. Our cox regression model showed that anticoagulant was the only predictor for hepatic decompensation and survival, with no other hepatic indicators.

Warfarin was chosen as an anticoagulant due to its low price and ease of administration. However, clinicians may be hesitant to use warfarin because CTPV cirrhotic patients with EHPVO always have esophagogastric varices. Several studies [12, 14, 15] have demonstrated that anticoagualtion treatment did not increase the bleeding risk of portal hypertension in patients with PVT. Our results demonstrated that the variceal bleeding rate was *lower* in the anticoagulation group than in the control group. Another disadvantage of warfarin treatment was the continuous adjustment of dosage by monitoring INR value, which reduced patient adherence. In clinical practice, the INR value of most patients will be stable at targeted value after a short time adjustment, and then patients need to monitor the INR value every month. There were no serious complications due to warfarin treatment. After a reduced dosage of warfarin, the most adverse event will diminish, such as gingival hemorrhage, urticaria, transit nausea and transit diarrhea.

As far as we know, this is the first study to evaluate the efficacy of warfarin for CTPV cirrhotic patients with EHPVO. However, there were several limitations in our research. Firstly, our study was one single-center retrospective study, with inevitable selection bias. Randomized controlled trials about this need to be further explored. Secondly, the assessment of changes in liver function after warfarin treatment was deficient, because our patients were always followed up at the local hospital instead of our center unless developed hepatic decompensation. Besides, the evaluation of esophagogastric varices by endoscopy was lacking.

## Conclusion

For CTPV cirrhotic patients with EHPVO, anticoagulation treatment could prevent hepatic decompensation and death, although had little effect on thrombosis recanalization.

## Supplementary Information

**Additional file 1:** **Supplement Table S1.** adverse events of patients in anticoagualtion group

## Data Availability

The datasets used and analyzed during the current study are available from the corresponding author on reasonable request.
