# Supplementary material for: Long-term efficacy and safety of anticoagulant for cavernous transformation of the portal vein cirrhotic patient with extrahepatic portal vein obstruction
Source: Thromb J. 2023 Jan 11;21:6. doi: 10.1186/s12959-023-00449-8 (PMC9832773; doi:10.1186/s12959-023-00449-8)
Supplement: Supplementary file 1 — Additional file 1: Supplement Table S1. adverse events of patients in anticoagualtion group [file 12959_2023_449_MOESM1_ESM.docx]

Supplement table S1: adverse events of patients in anticoagualtion group

| Events | Patients |
| --- | --- |
| Transit nausea | 3(6.5%) |
| Transit diarrhea | 2(4.3%) |
| Urticaria | 1(2.2%) |
| Gingival hemorrhage | 1(2.2%) |
